# Family and peer influences on adolescent psychological inflexibility: a regression mixture analysis

**DOI:** 10.3389/fpsyg.2025.1650930

**Published:** 2025-10-10

**Authors:** Lei Liu, Xiaomeng Hu, Mengyu Ma, Weilu Zhang, Biao Peng, Bixia Zhang, Xiaofang Luo

**Affiliations:** ^1^Public Administration School, Guangzhou University, Guangzhou, China; ^2^School of Marxism, Guangzhou University, Guangzhou, China; ^3^School of Public Administration, Hunan Normal University, Changsha, China; ^4^School of Marxism, Changsha Institute of Technology, Changsha, China; ^5^College of Music, Hunan Normal University, Changsha, China; ^6^Shenyang Jing’an Mental Health Hospital, Shengyang, China

**Keywords:** adolescent, family functioning, peer relationships, psychological inflexibility, regression mixture analysis

## Abstract

**Introduction:**

Adolescent mental health problems are closely linked to psychological inflexibility. Prior research has identified separate effects of family functioning and peer relationships on psychological inflexibility, but their combined effects (particularly from a person-centered perspective) remain underexplored. We use a person-centered regression mixture approach to jointly model family functioning and peer relationships and examine their joint associations with adolescent psychological inflexibility.

**Methods:**

The study surveyed 940 adolescents using the Family APGAR Index, the Peer Relationship Scale, and the Avoidance and Fusion Questionnaire for Youth to measure family functioning, peer relationships, and psychological inflexibility, respectively.

**Results:**

Through latent class analysis (LCA), six latent classes representing combined patterns of family functioning and peer relationships were identified. Adolescents exposed to both low family functioning and low peer relationships showed the highest psychological inflexibility, whereas those with high family functioning and high peer relationships showed the lowest. Regression mixture results indicated significant differences in psychological inflexibility across classes.

**Conclusion:**

These findings highlight the joint influence of family functioning and peer relationships on adolescent psychological inflexibility and suggest that interventions should concurrently target both environments to reduce inflexibility and improve mental health outcomes.

## Introduction

Since the global COVID-19 pandemic, the prevalence of psychological distress among adolescents has risen, becoming a significant public health issue (Benton et al., 2021). In recent years, researchers have increasingly focused on the role of psychological inflexibility in adolescents’ mental health. Psychological inflexibility, the opposite of psychological flexibility, refers to a behavior pattern in which individuals, when faced with thoughts, emotions, and other internal experiences, tend to avoid meaningful actions, which can negatively impact mental health (Levin et al., 2014). A growing body of evidence indicates that psychological inflexibility is significantly associated with depression, anxiety, and other mental health problems (Hernández-López et al., 2021; Pittman et al., 2024; Yao et al., 2023), and that interventions targeting psychological inflexibility can alleviate symptoms (Levin et al., 2014). However, existing studies have mostly treated psychological inflexibility as a predictor or mediator (e.g., between self-esteem and eating disorders, as shown by Koushiou et al., 2021) rather than examining its development as an outcome among adolescents. Against this backdrop, the present study is grounded in ecological systems theory and relational frame theory and focuses on two proximal microsystems central to adolescent development—family and peers (Bronfenbrenner and Morris, 1998; Hayes, 2004). These relational contexts shape adolescents’ “relational frames,” with adverse contexts more likely to engender rigid patterns and increase psychological inflexibility (Hayes et al., 2011).

On one hand, the family is the primary setting for adolescent development, and family functioning plays a crucial role in the onset and persistence of adolescent psychological and behavioral issues (Wang et al., 2022). Smilkstein identified five aspects of family functioning: adaptability, partnership, growth, affection, and resolve (Smilkstein, 1978). Adolescents in dysfunctional families are often influenced by harmful relational frames (e.g., “I’m unlovable”), which in turn lead to psychological inflexibility (Hayes et al., 2011). Previous studies have shown that authoritative parenting style (high warmth and control) and authoritarian parenting style (low warmth, high control) can predict psychological flexibility (An and Zhang, 2023; Williams et al., 2012). Emotional warmth from parents negatively predicts psychological inflexibility, while parental rejection and over-protection positively predict it (Peng et al., 2021).

On the other hand, as adolescence progresses, peer relationships play an increasingly important role in psychological development (Zhou and Zhou, 2021). Peer relationships refer to the interpersonal connections established and developed between individuals of similar age or psychological maturity (Rubin, 2011). Poor peer relationships create adverse contexts that increase experiential avoidance and cognitive fusion, both of which are core processes of psychological inflexibility (Hayes et al., 2011). Research has found that peer victimization damages cognitive flexibility (Liu et al., 2022), while adolescents’ experiential avoidance is related to histories of relational aggression and daily peer conflicts (Shea and Coyne, 2017; Xavier et al., 2018). Experiential avoidance and cognitive fusion are also linked to thwarted belongingness (Hapenny and Fergus, 2017). Additionally, online peer support programs can reduce psychological inflexibility and improve mental health outcomes (Grégoire et al., 2022).

Although prior studies have separately demonstrated the importance of family and peers, two gaps remain. First, previous research has lacked an integrated perspective. According to ecological systems theory, both family and peer environments are direct contexts for adolescent activities and interactions, directly influencing psychological development (Bronfenbrenner and Morris, 1998). Therefore, it is essential to consider the combined effects of family and peer relationships on psychological inflexibility. Second, most existing research is variable-centered, which overlooks the heterogeneity within the sample, thereby limiting the validity of the conclusions (Qiu, 2008). A variable-centered approach cannot fully capture the potential subgroups in family functioning and peer relationships or assess their unique contributions to psychological inflexibility. Thus, it is necessary to adopt a person-centered approach, using latent class analysis (LCA) to examine the different subgroups formed by the combined effects of family functioning and peer relationships, in order to better understand their influence on psychological inflexibility. This will also provide targeted directions for preventing adolescent mental health issues.

Accordingly, this study adopts a person-centered regression mixture framework: we first use LCA to identify joint types of family functioning and peer relationships, and then apply the Bolck-Croon-Hagenaars (BCH) method to compare differences across types in psychological inflexibility as a continuous outcome variable, in order to test their joint associations and inform stratified interventions. Based on this, we hypothesize the existence of distinct latent classes reflecting combined patterns of family and peer functioning, and that these classes will show significant differences in psychological inflexibility.

## Research methods

### Sample

This study selected a convenience sample of 1,032 secondary school students from three schools in Hunan and Guangdong provinces, China. Trained instructors initially explained the purpose, format, and anonymity of the questionnaire to the participants. Participants were asked again to ensure clarity on response requirements before they proceeded. They completed the questionnaire within a set time frame. All participants had signed an informed consent form before participation. In addition, participants were assured that all responses would be kept confidential and used solely for research purposes. All data were anonymized, and no personally identifiable information was collected. After discarding incomplete and patterned responses, 940 valid questionnaires were obtained. The valid response rate was 91.09%. The demographic breakdown was as follows: 417 males (44.36%) and 523 females (55.64%). The class distribution included 257 from the first year of junior high (27.34%), 235 from the second year (25.00%), 145 from the third year (15.43%), 79 from the first year of senior high (8.40%), 173 from the second year (18.40%), and 51 from the third year (5.43%). The average age was 14.22 years (*SD* = 1.77). This study received approval from the Academic Committee of Guangzhou University. All methods were conducted according to relevant guidelines and regulations.

### Measurements

#### Family functioning

We employed the Family APGAR Index developed by Smilkstein (1978). It uses a three-point Likert scale (often = 2, sometimes = 1, rarely = 0) across five dimensions: Adaptability, Partnership, Growth, Affection, and Resolve. This scale has shown good reliability and validity (Smilkstein et al., 1982; Yin et al., 2022). In this study, the Cronbach’s alpha was 0.89.

#### Peer relationships

The Peer Relationship Scale by Asher et al. (1984) was used, and its Chinese version has been validated (Zhang, 2008). It is scored on a four-point Likert scale from completely disagree to completely agree. The scale includes three dimensions: welcome, exclusion, and loneliness, with 16 items in total. Higher scores indicate better peer relationships. The Cronbach’s alpha in this study was 0.94.

#### Psychological inflexibility

The Avoidance and Fusion Questionnaire for Youth (AFQY) by Greco et al. (2008) was utilized. Its Chinese version, AFQ-Y8, is well-validated among Chinese adolescents (Chen et al., 2019). It consists of eight items, scored on a seven-point Likert scale from completely disagree to completely agree. Higher scores reflect greater psychological inflexibility. The Cronbach’s alpha for the AFQ-Y8 was 0.81 in this study.

### Statistical analysis

First, common method bias was tested using SPSS 24.0. Second, a latent class analysis of adolescents’ family functioning and peer relationships was conducted using Mplus Version 8.3. To reduce empty cells and improve the stability and interpretability of the model, response categories were recoded prior to the latent class analysis (three-point items were dichotomized into 0–1 vs. 2; four-point items were collapsed into 1–2 vs. 3–4). This approach has been widely recommended when ordered indicators have limited response frequencies and skewed distributions (Collins and Lanza, 2009; Nylund-Gibson and Choi, 2018). Although such recoding may reduce some variance information, the primary goal of latent class analysis is to identify underlying class patterns among groups rather than to capture subtle differences in continuous variables. Therefore, this treatment is considered unlikely to undermine the substantive validity of the class analysis results. To identify the best-fitting model, several fit statistics were used: Bayesian Information Criterion (BIC), Adjusted BIC (ABIC), Vuong-Lo–Mendell–Rubin Likelihood Ratio Test (VLMR LRT), Bootstrapped Likelihood Ratio Test (BLRT), and entropy. Entropy values range from 0 to 1, with values closer to 1 indicating more accurate classification (Lubke and Muthén, 2005). Finally, the BCH method was employed to predict psychological inflexibility using the latent class variables of family functioning and peer relationships. The BCH method is widely recognized for its robustness across various types of variables (Bakk et al., 2013).

## Results

### Common method bias test

The Harman’s single-factor test was employed to examine common method bias (Podsakoff et al., 2003). Exploratory factor analysis revealed four factors with eigenvalues greater than one, with the first factor accounting for 36.99% of the variance, which is below the critical threshold of 40%. This indicates that common method bias is not a significant concern in the present study.

### Descriptive statistics

Descriptive statistics are presented in Table 1. Peer relationships were positively correlated with family functioning and negatively correlated with psychological inflexibility (*p* < 0.01). Family functioning was negatively correlated with psychological inflexibility (*p* < 0.01).

**Table 1:** Correlation analysis results of each variable (*N* = 940).

Variable	M ± SD	1	2	3
1 peer relationship	49.39 ± 9.60	1		
2 family function	6.96 ± 2.66	0.50^**^	1	
3 psychological inflexibility	28.93 ± 9.24	−0.50^**^	−0.37^**^	1

**p < 0.01(two-tails).

### Latent class analysis

To explore the number of latent classes of family functioning and peer relationships among adolescents, we recoded the original scores of family functioning items from 0–1 to 0, and 2 to 1. For peer relationship items, original scores of 1–2 were recoded to 0, and 3–4 to 1. We estimated latent class models with 1–9 classes (see Table 2). The results showed that the model fit indices AIC, BIC, and ABIC decreased as the number of classes increased, with Entropy values all above 0.8. However, the LMR *p*-values were not significant for the 4-, 5-, 7-, 8-, and 9-class solutions. Therefore, we selected the 6-class model as the optimal fitting model.

**Table 2:** Model fit indices for LCA.

Model	*k*	AIC	BIC	ABIC	LMR	BLRT	Entropy	Class proportions
1	21	22493.017	22594.780	22528.086				
2	43	18888.952	19097.325	18960.760	0	0	0.901	0.670/0.330
3	65	17757.403	18072.385	17865.950	0	0	0.899	0.412/0.336/0.252
4	87	17420.379	17841.971	17565.665	0.227	0	0.879	0.357/0.201/0.298/0.144
5	109	17257.296	17785.497	17439.321	0.126	0	0.863	0.309/0.287/0.166/0.159/0.080
6	131	17079.559	17714.369	17298.322	0.031	0	0.849	0.289/0.230/0.135/0.169/0.089/0.087
7	153	17043.482	17784.902	17298.985	0.476	0	0.856	0.289/0.046/0.173/0.221/0.096/0.088/0.086
8	175	17001.506	17849.535	17293.747	0.240	0	0.851	0.321/0.067/0.156/0.090/0.068/0.104/0.106/0.086
9	197	16956.514	17911.152	17285.495	0.175	0	0.850	0.046/0.154/0.124/0.027/0.071/0.279/0.079/0.048/0.172

*k* = number of free parameters.

**Figure 1:**
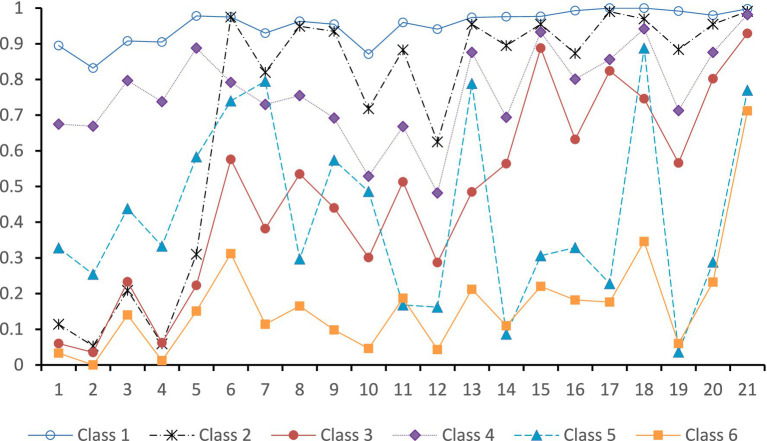
Conditional probabilities of 21 items for six latent classes of adolescent family functioning and peer relationships. Items 1–5 measure family functioning, items 6–10 measure welcome, items 11–16 measure loneliness, and items 17–21 measure exclusion. Lower conditional probabilities for family functioning items indicate lower family functioning. Lower conditional probabilities for welcome items indicate less welcome. Lower conditional probabilities for loneliness items indicate greater loneliness. Lower conditional probabilities for exclusion items indicate greater exclusion.

As shown in Figure 1, the conditional probability distributions of each class exhibited distinct differences, reflecting varied characteristics in peer relationships and family functioning. Class 1 adolescents showed conditional probabilities between 0.8 and 1 across all dimensions of family functioning and peer relationships. This indicates high levels in both family functioning and peer relationships, characterized by high family functioning, high welcome, low loneliness, and low exclusion Thus, Class 1 was labeled as the “High Family Functioning and High Peer Relationships” group, comprising 28.9% of the total sample. Class 2 adolescents had conditional probabilities between 0 and 0.3 for family functioning, while their peer relationship probabilities ranged from 0.6 to 1, slightly lower than Class 1. This suggests low family functioning but high peer relationships. Therefore, Class 2 was named the “Low Family Functioning and High Peer Relationships” group, accounting for 23.0% of the sample. Class 3 adolescents showed conditional probabilities between 0 and 0.3 for family functioning; for peer relationships, welcome ranged from 0.3 to 0.6, loneliness (except for item 15) from 0.5 to 0.6, and exclusion from 0.5 to 0.9. Considering these factors, this class was labeled as the “Low Family Functioning and Moderate Peer Relationships” group, representing 13.5% of the sample. Class 4 adolescents had conditional probabilities of 0.7–0.9 for family functioning, lower than Class 1 but higher than other classes. Their peer relationship probabilities ranged from 0.5 to 0.9, lower than Classes 1 and 2 but higher than the remaining classes. Thus, Class 4 was named the “Higher Family Functioning and Higher Peer Relationships” group, comprising 16.9% of the sample. Class 5 adolescents showed conditional probabilities between 0.2 and 0.6 for family functioning, indicating an overall moderate level. Their peer relationship probabilities were fluctuating but generally at a relatively low level (slightly higher than Class 6 but lower than other classes). Therefore, Class 5 was labeled as the “Moderate Family Functioning and Lower Peer Relationships” group, accounting for 8.9% of the sample. Class 6 adolescents exhibited the lowest conditional probabilities for both family functioning and peer relationships, representing 8.7% of the sample. Consequently, this class was named the “Low Family Functioning and Low Peer Relationships” group.

### Regression mixture model

Regression mixture models were constructed using different latent classes of adolescent family functioning and peer relationships as independent variables and psychological inflexibility as the outcome variable. The results are presented in Tables 3, 4. The differences in predicting psychological inflexibility among the six latent classes of adolescent family functioning and peer relationships were statistically significant (*χ*^2^ = 204.72, *p* < 0.001). The ranking of psychological inflexibility scores was as follows: Class 6 (*M* = 37.98) > Class 5 (*M* = 33.376) > Class 3 (*M* = 31.85) > Class 4 (*M* = 29.052) > Class 2 (*M* = 28.551) > Class 1 (*M* = 23.671).

**Table 3:** Descriptive statistics of psychological inflexibility indicators in different latent classes of adolescents.

Subgroup		Psychological inflexibility	
		*M*	SE
Class 1	High family functioning and high peer relationships	23.67	0.54
Class 2	Low family functioning and high peer relationships	28.55	0.62
Class 3	Low family functioning and moderate peer relationships	31.85	0.88
Class 4	Higher family functioning and higher peer relationships	29.05	0.76
Class 5	Moderate family functioning and lower peer relationships	33.38	1.06
Class 6	Low family functioning and low peer relationships	37.98	1.12

**Table 4:** Differences in psychological inflexibility indicators among different latent classes of adolescents.

Subgroup	Psychological inflexibility		Subgroup	Psychological inflexibility	
	*χ* ^2^	*p*		*χ* ^2^	*p*
Class 1 vs. 2	34.567	0.000	Class 2 vs. 6	54.545	0
Class 1 vs. 3	63.889	0.000	Class 3 vs. 4	5.611	0.018
Class 1 vs. 4	29.775	0	Class 3 vs. 5	1.131	0.288
Class 1 vs. 5	66.678	0	Class 3 vs. 6	17.646	0
Class 1 vs. 6	133.238	0	Class 4 vs. 5	10.654	0.001
Class 2 vs. 3	8.4	0.004	Class 4 vs. 6	43.912	0
Class 2 vs. 4	0.246	0.62	Class 5 vs. 6	8.251	0.004
Class 2 vs. 5	15.45	0			

Specific comparisons revealed that Class 6 had significantly higher psychological inflexibility scores than all other categories. There was no significant difference in psychological inflexibility scores between Class 5 and Class 3 (*χ*^2^ = 1.131, *p* = 0.29), but both categories differed significantly from the others. No significant difference was found between Class 1 and Class 2 (*χ*^2^ = 0.246, *p* = 0.620), while these two categories showed significant differences from all other categories. Class 1 had significantly lower psychological inflexibility scores than all other categories.

From a practical perspective, these differences are also meaningful in magnitude. For instance, adolescents in Class 6 scored more than 14 points higher in psychological inflexibility compared to those in Class 1, representing a substantial disadvantage for adolescents simultaneously experiencing poor family functioning and weak peer relationships. Similarly, although Classes 5 and 3 did not differ significantly from each other, both scored 8–10 points higher than Class 1, suggesting that even moderate difficulties in either family or peer relationships are linked to noticeable increases in psychological inflexibility. Conversely, the similarity between Class 1 and Class 2 indicates that strong peer relationships may buffer the negative effects of low family functioning.

## Discussion

Previous studies have demonstrated that family environment and peer relationships play crucial roles in the development of psychological inflexibility among adolescents (Peng et al., 2021; Shea and Coyne, 2017; Williams et al., 2012; Xavier et al., 2018). However, prior research has largely focused on parenting styles or single dimensions, lacking an in-depth exploration of overall family functioning and the combined effects of family and peer relationships. Additionally, existing studies have primarily employed variable-centered approaches, overlooking within-sample heterogeneity and failing to fully reveal the impact of different combinations of family functioning and peer relationships on adolescent psychological inflexibility. Therefore, this study adopted a person-centered approach to examine the joint patterns of family functioning and peer relationships and their influence on adolescent psychological inflexibility, providing both an integrated perspective that extends beyond single-context studies and methodological innovation that reveals heterogeneous subgroup patterns and pathways that variable-centered approaches would have obscured.

### Latent classes of adolescent family functioning and peer relationships

Family and peer environments are the immediate contexts for adolescent activities and interactions, exerting the most critical influence on adolescent psychological development (Bronfenbrenner and Morris, 1998). Through LCA, this study identified six latent classes of adolescents in terms of family functioning and peer relationships, which exhibited significant differences across various dimensions of family functioning and peer relationships. Unlike traditional single-dimension analyses, this study integrated five key aspects of family functioning (adaptability, partnership, growth, affection, and resolve) and three dimensions of peer relationships (acceptance, loneliness, and rejection), providing a comprehensive understanding of how family functioning and peer relationships influence adolescent psychological development and psychological inflexibility.

Family functioning has a significant impact on the quality of adolescent peer relationships. Children’s early social interaction behaviors are primarily learned from family members and continue to influence their peer interaction patterns during adolescence (Boele et al., 2019; Yin et al., 2022). Good family functioning provides adolescents with emotional support and communication skills, enabling them to perform better in peer interactions (Nie et al., 2020; Shen, 2020). Accordingly, this study did not identify a “high family functioning and low peer relationships” group; rather, it identified the groups with high family functioning and high peer relationships, as well as higher family functioning and higher peer relationships.

Adolescents with moderate family functioning may receive partial emotional support from their families, but such support may be insufficient to help them effectively establish and maintain positive peer relationships. Their friendships may appear fragile and easily disrupted, often marked by conflict and ambivalence. As a result, this study identified a group characterized by moderate family functioning and relatively low peer relationships.

When family functioning is low, adolescents may rely more heavily on peer relationships to obtain emotional support and a sense of belonging (Rubin et al., 2004). This compensatory mechanism may motivate them to invest greater effort in building and maintaining high-quality peer relationships, leading to the patterns of “low family functioning and moderate peer relationships” and “low family functioning and high peer relationships.” However, for a small minority of adolescents (8.7%), the influence of low family functioning prevents the formation of secure attachment, leading to a lack of security and trust in interpersonal interactions, resulting in poor peer relationships (Peng and Pan, 2023). Consequently, this manifests as the low family functioning and low peer relationships type.

Impact of Combined Patterns of Family Functioning and Peer Relationships on Adolescent Psychological Inflexibility.

Previous studies have confirmed the significant individual impacts of family functioning and peer relationships on the development of psychological inflexibility (Grégoire et al., 2022; Williams et al., 2012). However, research on the combined effects of family functioning and peer relationships on adolescent psychological inflexibility has been lacking. The present study demonstrates that six distinct combined patterns of family functioning and peer relationships significantly influence the degree of psychological inflexibility. Specifically, adolescents in the low family functioning and low peer relationships group exhibited the highest levels of psychological inflexibility. Unable to obtain emotional support from their families or social recognition through peer interactions, this dual disadvantage profoundly negatively impacts their psychological health development. Relational Frame Theory posits that adverse contexts can lead to harmful “relational frames,” and adherence to these frames exacerbates the development of psychological inflexibility (Hayes et al., 2011). Conversely, adolescents in the high family functioning and high peer relationships group demonstrated the lowest levels of psychological inflexibility. This finding aligns with previous research, indicating that positive family functioning and peer relationships have a synergistic effect on adolescent psychological health development (Williams and Anthony, 2015). Although relational frame theory and functional contextualism explain how adverse contexts can lead to higher levels of psychological inflexibility, they do not adequately account for how positive environments, such as supportive family functioning and strong peer relationships, reduce psychological inflexibility. In fact, an increasing body of research has found that positive family functioning and peer relationships exert beneficial effects by enhancing adolescents’ self-esteem (Huang et al., 2022; Wang et al., 2017). High self-esteem, in turn, can mitigate the impact of negative self-concept (one of the six core processes of psychological inflexibility), thereby reducing psychological inflexibility (Peng et al., 2021).

Furthermore, the study found that the moderate family functioning and lower peer relationships group exhibited slightly higher levels of psychological inflexibility than the low family functioning and moderate peer relationships group, though the difference was not statistically significant. Similarly, the low family functioning and high peer relationships group showed slightly lower levels of psychological inflexibility than the higher family functioning and higher peer relationships group, but again, the difference was not significant. This suggests that peer relationships may, in certain developmental contexts, exert a stronger influence on psychological inflexibility than family functioning. Previous research has also indicated that adolescents often prioritize peer relationships over parent–child relationships, and peer influences may, under some circumstances, become more salient than parental influences (Zhang et al., 2021). Particularly in cases of poor family functioning, positive peer interactions can partially compensate for the negative effects of family dysfunction (Criss et al., 2002). Positive peer relationships provide emotional support and a sense of belonging, helping adolescents solve practical problems rather than resorting to experiential avoidance strategies (Grégoire et al., 2022; Hapenny and Fergus, 2017; Xavier et al., 2018), thereby reducing psychological inflexibility. Additionally, positive peer relationships can lower psychological inflexibility by promoting adolescent self-esteem (Peng et al., 2021). Nevertheless, low family functioning still restricts psychological flexibility in these adolescents. As a result, the low family functioning and high peer relationship group showed higher levels of psychological inflexibility than the high family functioning and high peer relationship group. This indicates that even in the presence of positive peer interactions, the absence of family functioning can still positively influence adolescent psychological inflexibility.

### Implications

This study reveals the significant impact of combined patterns of family functioning and peer relationships on adolescent psychological inflexibility, particularly highlighting the highest levels of inflexibility under conditions of low family functioning and poor peer relationships. These findings provide multi-level, practical implications for families, schools, communities, and policymakers.

Firstly, at the policy and regional coordination level, it is recommended to integrate family-peer collaborative interventions into regional public mental health service systems, thereby building replicable and sustainable support mechanisms. Regional youth mental growth centers may be established to integrate resources from education, health, community, and social organizations, operating under a model of “dedicated management, professional support, and specialized programs.” Local governments and public funding should provide basic support for low-cost interventions. In resource-limited areas, priority should be given to cultivating “peer experts” and establishing online support platforms. Low-cost approaches such as parent mutual-aid groups, training teachers as mental health facilitators, and disseminating psychoeducational content via video channels can be implemented to expand coverage and share experiences in regional mental health education.

Secondly, at the family level, improving family functioning is an important pathway to reducing adolescent psychological inflexibility. It is suggested to rely on regional parent schools and online parenting programs to provide systematic training and support for parents, with a focus on enhancing five key dimensions of family functioning: (1) Adaptability—helping families adjust to changing circumstances; (2) Partnership—promoting collaborative problem-solving and decision-making; (3) Growth—encouraging personal development of family members; (4) Affection—fostering emotional bonds and expression of care; and (5) Resolve—improving commitment to family responsibilities and time management. Parents should also be encouraged to participate in mutual-aid volunteer services, achieving a “role reversal” from being recipients of help to becoming helpers themselves, thereby strengthening their agency and capacity for both self-help and helping others.

Thirdly, at the school level, a “dual-track support” system should be built, simultaneously enhancing family functioning and strengthening peer relationships. Schools can adopt group activities, peer psychological support programs, and social skills training to help adolescents develop positive and healthy peer relationships, compensating for deficiencies in family functioning. For rural and resource-limited schools, special efforts should be made to cultivate in-school mental health backbone teachers, promote initiatives such as the “growth partner” program and classroom mental health monitors, and carry out diverse, low-threshold extracurricular activities to enhance students’ sense of belonging and self-worth.

Finally, the importance of family-school-community collaborative intervention must be emphasized. Schools and communities should cooperate to launch comprehensive service projects integrating family counseling, parent–child workshops, and peer support groups, thereby creating a micro-system of “family-school-community” collaboration in mental health. By combining offline services with online courses, both foundational family education principles and individualized guidance can be delivered. This provides adolescents with a comprehensive and multidimensional emotional support network, effectively improving psychological flexibility and reducing the risk of psychological inflexibility.

### Limitations

Despite the valuable findings of this study, several limitations should be noted. Firstly, the cross-sectional design precludes the establishment of causal relationships between family functioning, peer relationships, and adolescent psychological inflexibility, and does not allow for the examination of long-term effects. Future studies should employ longitudinal research designs and, where feasible, incorporate observational or physiological measures (e.g., cortisol levels, heart rate variability) to capture dynamic and objective indicators of adaptation. Secondly, this study primarily relied on self-report questionnaires, which may be subject to participant bias, including social desirability bias, especially among adolescents within collectivist cultural contexts such as China. Future studies should combine multi-informant reports (e.g., parents, teachers, peers) and behavioral observations to enhance the validity and depth of the data. Thirdly, the use of a school-based convenience sample from three schools in two provinces may limit the generalizability of the findings. In particular, the influence of culturally specific factors—such as collectivist family structures, intergenerational dynamics, and emphasis on academic achievement—may affect the manifestation of family functioning and peer relationships within the Chinese context. Caution is advised when generalizing these results to other cultural or educational settings. Future studies should adopt multi-site, probability-based sampling strategies and examine the cultural and contextual factors that may moderate the observed relationships.

## Conclusion

Employing a person-centered approach, this study demonstrates that adolescent psychological inflexibility is shaped not in isolation by either family or peer environments, but through their combined patterns. We identified six distinct configurations of family functioning and peer relationships, among which adolescents experiencing low levels in both domains showed the highest psychological inflexibility. These findings advance current understanding by highlighting the interactive nature of social contexts in adolescent development. Consequently, future interventions should adopt an integrated approach, simultaneously targeting family functioning and peer relationships to effectively promote psychological flexibility and mental health.

## Data Availability

The raw data supporting the conclusions of this article will be made available by the authors, without undue reservation.
